# The Microvillar and Solitary Chemosensory Cells as the Novel Targets of Infection of SARS-CoV-2 in Syrian Golden Hamsters

**DOI:** 10.3390/v13081653

**Published:** 2021-08-20

**Authors:** Jin-Seok Seo, Sun-Woo Yoon, Seung-Hyeon Hwang, Sung-Min Nam, Sang-Soep Nahm, Jei-Hyun Jeong, Jiho Lee, Ha-Na Youn, Jun-Beom Kim, Woosuk Kim

**Affiliations:** 1 Department of Anatomy, College of Veterinary Medicine, Konkuk Unitversity, Seoul 05030, Korea; phoenix0718@konkuk.ac.kr (J.-S.S.); dbstjsdn99@konkuk.ac.kr (S.-W.Y.); akider501@konkuk.ac.kr (S.-H.H.); ssnahm@konkuk.ac.kr (S.-S.N.); 2 Jesaeng-Euise Clinical Anatomy Center, Department of Anatomy, School of Medicine, Wonkwang University, Iksan 54538, Korea; namvet1@wku.ac.kr; 3 Veterinary Science Research Institute, College of Veterinary Medicine, Konkuk Unitversity, Seoul 05030, Korea; 4 Department of Avian Diseases Laboratory, College of Veterinary Medicine, Konkuk University, Seoul 05030, Korea; nar21ss@hanmail.net (J.-H.J.); jiho5933@gmail.com (J.L.); 5 KCAV Co., Ltd., Seoul 05030, Korea; yellow0891@hanmail.net (H.-N.Y.); kjb900809@naver.com (J.-B.K.)

**Keywords:** SARS-CoV-2, olfactory system, microvillar cell, solitary chemosensory cell, regeneration

## Abstract

Patients infected with severe acute respiratory syndrome coronavirus 2 (SARS-CoV-2), the causative agent of coronavirus disease 2019, suffer from respiratory and non-respiratory symptoms. Among these symptoms, the loss of smell has attracted considerable attention. The objectives of this study were to determine which cells are infected, what happens in the olfactory system after viral infection, and how these pathologic changes contribute to olfactory loss. For this purpose, Syrian golden hamsters were used. First, we verified the olfactory structures in the nasal cavity of Syrian golden hamsters, namely the main olfactory epithelium, the vomeronasal organ, and their cellular components. Second, we found angiotensin-converting enzyme 2 expression, a receptor protein of SARS-CoV-2, in both structures and infections of supporting, microvillar, and solitary chemosensory cells. Third, we observed pathological changes in the infected epithelium, including reduced thickness of the mucus layer, detached epithelia, indistinct layers of epithelia, infiltration of inflammatory cells, and apoptotic cells in the overall layers. We concluded that a structurally and functionally altered microenvironment influences olfactory function. We observed the regeneration of the damaged epithelium, and found multilayers of basal cells, indicating that they were activated and proliferating to reconstitute the injured epithelium.

## 1. Introduction

Severe acute respiratory syndrome coronavirus 2 (SARS-CoV-2) is the causative agent of coronavirus disease 2019 (COVID-19). Patients with COVID-19 have various symptoms. The Centers for Disease Control and Prevention (CDC) updated the list of symptoms of the disease in 2020 to include fever or chills, cough, shortness of breath, difficulty breathing, fatigue, muscle or body aches, headache, new loss of taste or smell, sore throat, congestion or runny nose, nausea or vomiting, and diarrhea [1]. Among these signs, loss of smell has attracted attention because it has been reported in mild or even asymptomatic cases, and it is useful as a predictive method [2,3,4,5]. Loss of smell induced by SARS-CoV-2 is unique, in that it has a sudden onset, rather short duration, and rapid resolution [6]. Most olfactory dysfunction resolves within approximately two weeks [2,6].

Clinical symptoms are closely related to virus-infected cells. To infect host cells, vi-ruses need to bind to receptors expressed on the cell membrane of host cells. In the case of SARS-CoV-2, the viral spike (S) protein binds to angiotensin-converting enzyme 2 (ACE2) after being primed by transmembrane protease, serine 2 (TMPRSS2) [7]. Therefore, host cells expressing both ACE2 and TMPRSS2 may be targets of the virus. ACE2 is expressed in enterocytes, renal tubules, the gall bladder, cardiomyocytes, male reproductive cells, placental trophoblasts, ductal cells, the eye, vasculature, and type II alveolar cells in humans [8,9]. Additionally, studies have investigated ACE2 and TMPRSS2 expression in the nasal cavity because olfactory dysfunctions found in patients with COVID-19 might be related to infection in the nasal cavity [10,11,12,13,14,15,16].

Most of the human nasal cavity is lined by the non-sensory mucosa. Only a small portion, called the olfactory cleft, is occupied by sensory neuroepithelium and is responsible for odor perception. The neuroepithelium is mainly occupied by olfactory sensory neurons (OSNs). They extend their dendrites to the nasal cavity. At its distal terminal end, dendrites are modified to form dendritic knobs or olfactory vesicles, which extend non-motile cilia to the olfactory cavity. Olfactory cilia of OSNs have numerous copies of a particular odor-receptor molecule. If odiferous substances bind to odor-receptor molecules, OSNs are stimulated. They send information to the olfactory bulb by their axons penetrating the basal lamina and passing through the cribriform plate for further processing of information. In addition to OSNs, the cells constituting the olfactory epithelium include supporting cells (SCs), basal cells (BCs), microvillar cells (MCs), and ductal cells. These cellular components structurally and functionally influence each other. Their interactions are important for olfaction [17,18].

Until now, although many studies have focused on the nasal cavity, opinions have been divided regarding which cell type expresses receptor proteins and is infected by the virus. These studies have also mainly concentrated upon OSNs, which are directly related with olfaction. However, other cells that constitute the olfactory system should not be overlooked. An accurate understanding is required because the mechanism of olfactory dysfunction can be changed, depending on which cell type is infected. Therefore, in this study, we infected the Syrian golden hamster (*Mesocricetus auratus*) with SARS-CoV-2 and observed histopathologic changes in the nasal cavity after infection. Syrian hamsters were used as animal models of SARS-CoV-2 infection because experiments using these hamsters can be completed quickly and cost effectively [10,19,20,21,22]. Here, we determined the distribution of ACE2 and virus-infected cellular components of olfactory structures. Furthermore, we tried to explain the possible causes of olfactory dysfunction by combining our findings with those of previous studies. Afterward, we observed the results of the damaged olfactory system and its underlying mechanism, whether it regenerated properly or not.

## 2. Materials and Methods

### 2.1. Viruses and Animals

SARS-CoV-2 isolated from throat swabs of patients with confirmed COVID-19 was purchased from the National Culture Collection for Pathogens (NCCP) (NCCP No. 43326 Human coronavirus (BetaCoV/Korea/KCD203/2020)). The virus was subcultured in Vero E6 cells and stored at −80 °C until use.

The experimental design of the present study was approved by the Institutional Animal Care and Use Committee of Konkuk University (KU20163). Male Syrian golden hamsters (12-week-old) were purchased from Central Lab Animal Inc. and housed under adequate temperature (22 ± 2 °C) and humidity (60 ± 5%) at the Institute of Biomedical Science and Technology of Konkuk University (Animal Biosafety Level (BSL) 3). The hamsters were acclimatized for one week and then used for the experiments. After acclimatization, hamsters (*n* = 2 per group) were randomly divided into two groups. One group, the infected group, was infected with a 10^5^ TCID_50_ (50% tissue culture infective dose) of SARS-CoV-2 diluted in 200 μL of phosphate buffered saline (PBS) by nasal inoculation. The mock-infected control group received only PBS. All experiments related to the virus were performed in a BSL3 facility.

### 2.2. Tissue Preparation and Histopathological Assessment

For histological assessment, the hamsters were euthanized by inhalation of carbon dioxide four days post-infection (dpi). Whole heads of the hamsters were dissected. The mandible, scalp, muscles, calvaria, and brain were removed to facilitate penetration of the fixative, and the rest of the heads were fixed in 10% neutral-buffered formalin for three days at 4 °C. After fixation, they were decalcified in MoL-decalcifier solution (Milestone Medical, Sorisole, BG, Italy) for three weeks at room temperature (25 °C) with gentle shaking. The nasal cavity was then cut into four equal parts, which were processed, embedded in paraffin blocks, cut into 5 µm thick sections, and attached to silane-coated slides (MUTO Pure Chemicals, Tokyo, Japan).

Alcian blue staining was performed using the Alcian blue pH 2.5 staining kit (BBC Biochemical, Mt Vernon, WA, USA). In brief, sections were deparaffinized and rehydrated in xylene and graded alcohols. Sections were stained with Alcian blue solution for 15 min in a 37 °C water bath. After washing, the sections were counterstained with nuclear fast red for 5 min. Sections were dehydrated and cleared in graded alcohol and xylene. Finally, the sections were mounted with a toluene-based mounting medium (Thermo Scientific, Waltham, MA, USA).

### 2.3. Immunostaining

For immunohistochemistry, rehydrated sections were pretreated using the heat-induced epitope retrieval method in a microwave with Tris-EDTA solution (pH = 9). Subsequently, they were quenched with 3% hydrogen peroxide and blocked with normal serum from the same host of the secondary antibodies (Vector Laboratories, Burlingame, CA, USA) to prevent non-specific antibody binding. Next, the sections were incubated with primary antibodies diluted in blocking solution overnight at 4 °C. The primary antibodies used in this study and their targets are listed in Table 1. After washing, the sections were incubated with biotinylated secondary antibodies (Table 2), followed by horseradish peroxidase-conjugated streptavidin. 3,3′-diaminobenzidine tetrachloride (DAB; Tokyo Chemical Industry, Tokyo, Japan) was used as a chromogen to visualize the sections. The sections were dehydrated in alcohol and xylene after counterstaining with methyl green (Sigma-Aldrich, St. Louis, MO, USA) and then mounted with toluene-based mounting medium.

For dual immunofluorescence, sections were treated using the same procedure as described for DAB immunostaining. After incubation with primary antibodies, the sections were exposed to a mixture of fluorochrome-conjugated immunoglobulin at room temperature (Table 2). Subsequently, they were washed and mounted with antifade mounting media containing DAPI (Vector, Laboratories, Burlingame, CA, USA).

### 2.4. Histopathological Assessments and Statistical Analysis

All sections were examined and captured with the Olympus microscope BX51 equipped with the camera DP 74 using cellSens standard software (Olympus, Tokyo, Japan). Images were further processed using Fiji ImageJ software version 1.52p (National Institutes of Health, Bethesda, MD, USA) by adjusting brightness, contrast, and color balance. To analyze the histopathological changes in Alcian blue staining, five randomly selected images, including of the nasal septum, endoturbinates, and ectoturbinates, were observed in high magnification microscope fields (×400). They were assessed in the following three aspects: mucus thickness, mucus cover, and epithelial damage. To assess mucus thickness, images were given a score of 0–3 as follows: thick (score 3), medium (score 2), thin (score 1), and unobservable (score 0) mucus. To assess mucus cover, images were given a score of 0–3 as follows: mostly (75–100%, score 3), moderately (50–75%, score 2), mildly (25–50%, score 1), and minimally (0–25%, score 0) covering the epithelium. To assess epithelial damage, images were given a score of 0–3 as follows: minimal (three distinct layers with no detachment, score 3), mild (three distinct layers but with some detached epithelia, score 2), moderate (absence of three distinct layers and presence of epithelial detachment, score 1), and severe (most epithelia are detached and the basal lamina is exposed, score 0) damage. The scoring was performed by three investigators who were blinded to the study. The Mann–Whitney U test was applied to analyze these data using Prism software version 8.2.1 (GraphPad, San Diego, CA, USA).

## 3. Results

### 3.1. Components of the Olfactory Epithelium of the Hamsters

**Figure 1:**
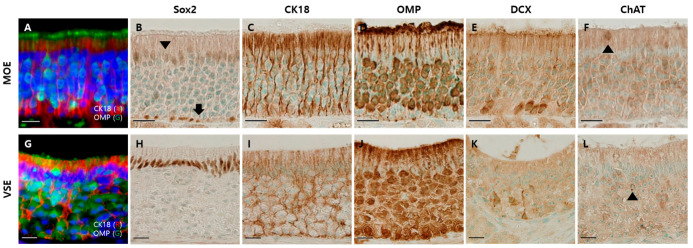
Olfactory system in the nasal cavity of the hamsters. Scale bars = 20 µm. (**A**) Double immunofluorescent labeling of OMP and CK18 in the main olfactory epithelium (MOE), which are markers for mature olfactory sensory neurons (mOSNs) and supporting cells (SCs), respectively; (**B**) DAB staining with the antibody to Sox2 in MOE: nuclei of SCs (arrowhead) and basal cells (BCs, arrow); (**C**) DAB staining with the antibody to CK18 in MOE: cytoplasm and feet of SCs; (**D**) DAB staining with the antibody to OMP in MOE: mOSNs; (**E**) DAB staining with DCX antibody in MOE: immature olfactory sensory neurons; (**F**) DAB staining with the antibody to ChAT in MOE: microvillar cells (arrowhead); (**G**) double immunofluorescent labeling of OMP and CK18 in vomeronasal sensory epithelium (VSE); (**H**) DAB staining with the antibody to Sox2 in VSE: nuclei of SCs; (**I**) DAB staining with the antibody to CK18 in VSE: cytoplasm and feet of SCs; (**J**) DAB staining with OMP antibody in VSE: mature vomeronasal sensory neurons; (**K**) DAB staining with DCX antibody in VSE: immature vomeronasal sensory neurons; (**L**) DAB staining with ChAT antibody in VSE: solitary chemosensory cells (arrowhead).

Before identifying SARS-CoV-2 infection in the olfactory system of the Syrian hamsters, we determined the cellular components of the olfactory system based on previous studies [17,18,23,24,25]. We investigated the representative olfactory system in nasal passages: the main olfactory epithelium (MOE) and the vomeronasal organ (VNO) [26]. There were three distinct layers in the MOE (Figure 1A). The nuclei of SRY-box 2 (Sox2)-positive cells were found in both the apical and basal layers (Figure 1B). Cytokeratin 18 (CK18)-positive cells spanned the whole MOE, and their cytoplasm was located in the apical layer (Figure 1C). The cytoplasm of olfactory marker protein (OMP)-positive cells occupied most of the intermediate region. Their dendrites passed through the apical layer and were exposed to the nasal cavity (Figure 1D). The cytoplasm of doublecortin (DCX)-positive cells was also in the intermediate region, but it was relatively below the cytoplasm of OMP-positive cells (Figure 1E). The choline acetyltransferase (ChAT)-positive cells were relatively few, and mainly found in the apical layer (Figure 1F).

The VNO has a vomeronasal sensory epithelium (VSE) and non-sensory epithelium (NSE) [26,27]. The structure of the VSE was similar to that of MOE, but there were several differences between MOE and VSE (Figure 1G). The nuclei of Sox2-positive cells were in the apical layer but not in the basal layer of the VSE (Figure 1H). The CK18-positive cells spanned the VSE (Figure 1I). The cytoplasm of OMP-positive cells occupied a major intermediate region throughout the VSE (Figure 1J). The cytoplasm of DCX-positive cells was mostly located in the transitional zone between NSE and VSE (Figure 1K). There were also several ChAT-positive cells in the VSE (Figure 1L).

### 3.2. SARS-CoV-2 Infection in the Olfactory Epithelium

**Figure 2:**
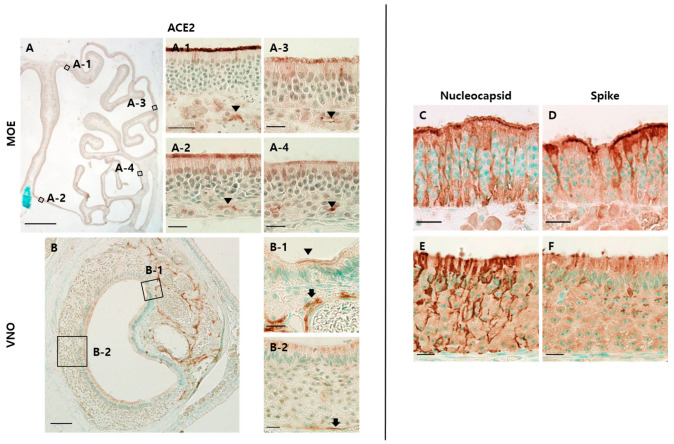
SARS-CoV-2 infections in the olfactory system. The scale bar in (**A**) is 1 mm, in (**D**) is 100 μm, and in all others is 20 μm. (**A**) DAB staining with the antibody to ACE2 in dorsomedial (**A-1**), ventromedial (**A-2**), dorsolateral (**A-3**), and ventrolateral (**A-4**) MOE of the mock group: ACE2 was expressed on the apical surface of the epithelium and luminal surface of Bowman’s glands (arrowhead). In the lateral region, ACE2 expression on the apical surface becomes weak and sparse; (**B**) DAB staining with the antibody to ACE2 in the non-sensory epithelium (NSE) and VSE: ACE2 was expressed on some of the apical surface of NSE and cavernous tissues (**B-1**). ACE2 expression was also observed in the apical portion of the VSE, intraepithelial capillaries, and blood vessel walls just below the epithelium (**B-2**); (**C**,**D**) DAB staining with the antibody to nucleocapsid (N) and spike (S) protein in the MOE of the infected group. Both N and S protein were expressed on the epithelial cells spanning the whole layer of the MOE; (**E**,**F**) DAB staining with the antibody to N and S protein in VSE. N protein was expressed on the whole height of the VSE, whereas S protein was expressed on the perinuclear region and apical portion of the VSE.

**Figure 3:**
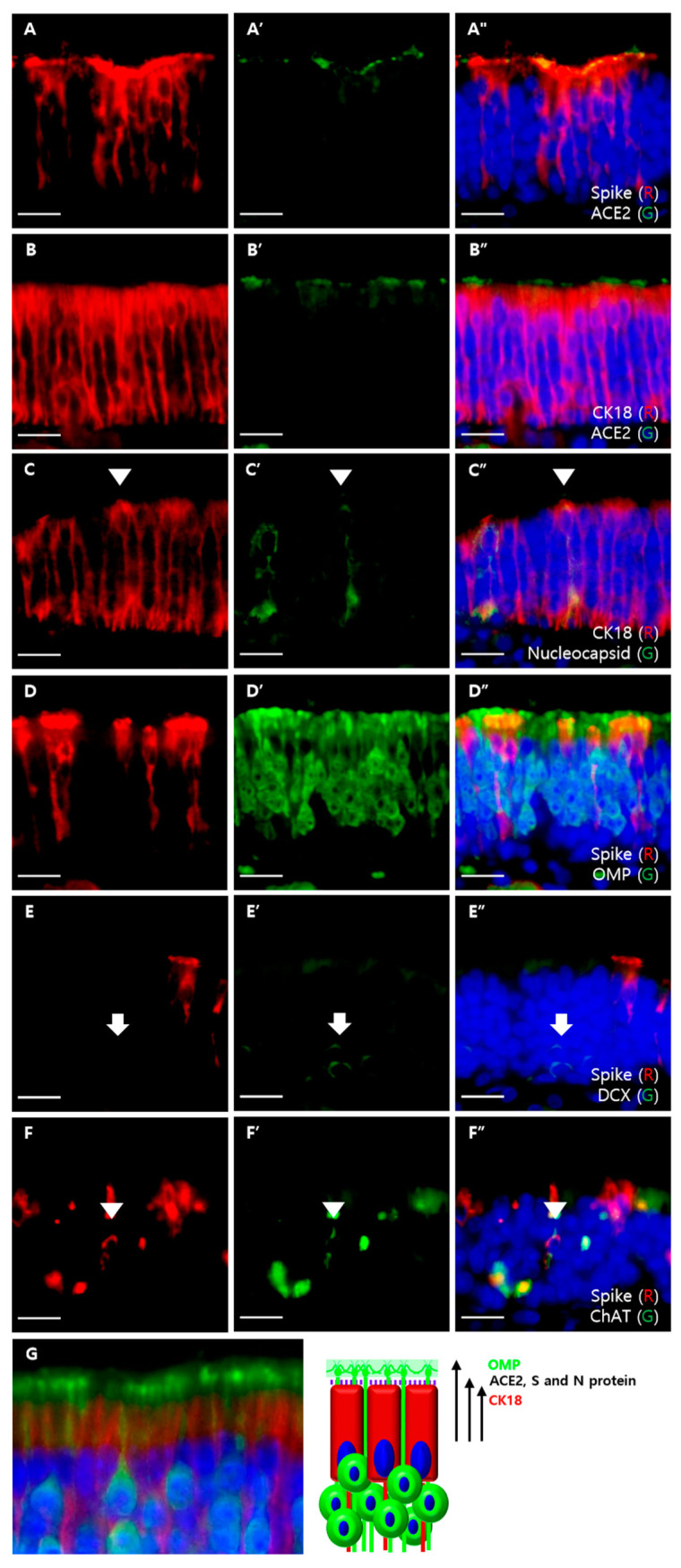
Double immunofluorescent labeling of MOE. All sections were counterstained with DAPI (blue). Scale bars = 20 μm. (**A**) Colocalization of the S protein (red) and ACE2 (green). The apical portion of the S protein and ACE2 had similar levels; (**B**) colocalization of CK18 (red) and ACE2 (green). The apical portions of CK18-positive cells were located lower than the levels of ACE2 expression; (**C**) colocalization of CK18 (red) and N proteins (green). Some CK18-positive cells were colocalized with the N protein (arrowhead); (**D**) colocalization of the S protein (red) and OMP (green). There was no colocalization between the S protein and OMP. Apical portions of S protein-positive cells were lower than apical portions of OMP-positive cells; (**E**) colocalization of the S protein (red) and DCX (green). There was no colocalization between the S protein and DCX (arrow); (**F**) colocalization of the S protein (red) and ChAT (green). Some ChAT-positive cells were colocalized with the S protein (arrowhead); (**G**) cropped images from Figure 1A that label CK18-positive SCs (red) and OMP-positive mOSNs (green), which are shown on the left, and a schematic diagram of ACE2-expressing cells is shown on the right. Using the modified sandwich method, we conclude that ACE2 is expressed on the cell membrane of SCs, which is not labeled by CK18 and is below the densely labeled OMP-positive dendritic knob.

**Figure 4:**
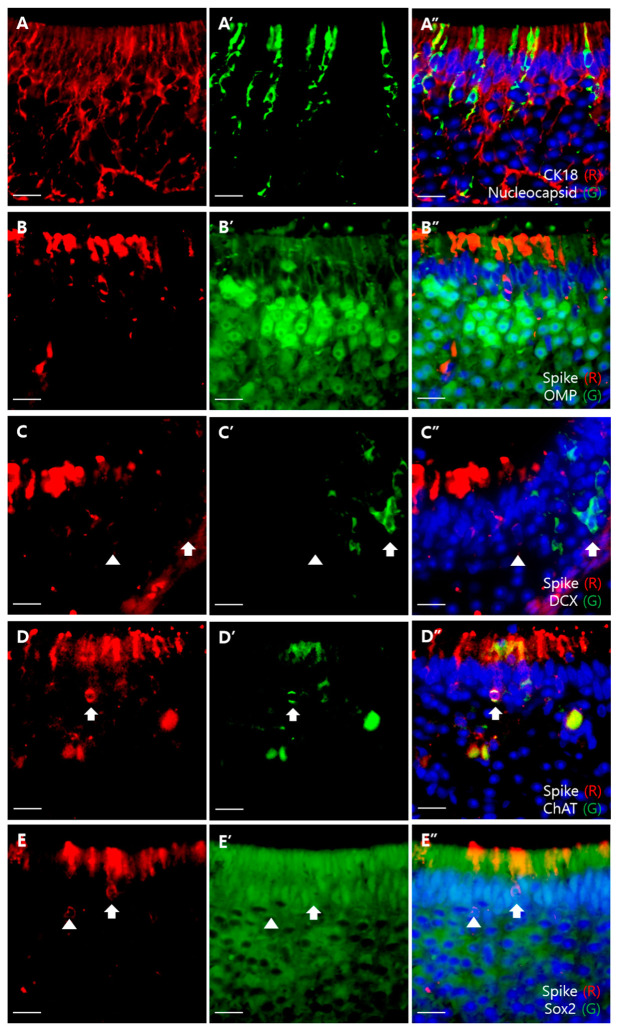
Double immunofluorescent labeling of the VSE. Scale bars = 20 μm. (**A**) Colocalization of CK18 (red) and nucleocapsid (green). Some CK18-positive cells colocalized with N protein; (**B**) colocalization of the S protein (red) and OMP (green). There was no colocalization between the S protein and OMP; (**C**) colocalization of the S protein (red) and DCX (green). There was no colocalization between the S protein and DCX; (**D**) colocalization of the S protein (red) and ChAT (green) The ChAT-positive cell was colocalized with the S protein; (**E**) colocalization of the S protein (red) and Sox2 (green). The S protein-positive cells encircle Sox2-positive nuclei (arrow), but there are also S protein-positive cells encircling the Sox2-negative nuclei (arrowhead).

We identified ACE2 expression in both MOE and VNO (Figure 2A,D). In MOE, ACE2 was expressed on the luminal surface of the epithelium and Bowman’s glands in all places. The expression on the luminal surface was strong in the dorsomedial MOE (Figure 2A-1) and weak in the ventromedial MOE (Figure 2A-2). In the lateral MOE, ACE2 expression became weak and sparse (Figure 2A-3,A-4). In VNO, ACE2 was expressed strongly in the cavernous tissue near the NSE and weakly in the apical portion of the NSE (Figure 2B-1). It was also found in the apical portion of the VSE and blood vessels near the basal lamina and intraepithelial capillaries (Figure 2B-2).

Next, we identified SARS-CoV-2 infection in the olfactory epithelium by staining S and nucleocapsid (N) proteins. We found both proteins in the MOE and VSE. In the MOE, both N and S proteins were expressed on cells spanning the whole layer of the epithelium (Figure 2C,D). In VSE, N protein was expressed on the whole height of the epithelia, but S protein was expressed only in the luminal portion and perikarya located in the apical layer (Figure 2E,F).

To determine which cell type expresses the ACE2 protein and is infected by SARS-CoV-2, we performed double immunofluorescence staining using the sandwich method that was slightly modified from a previous study [11]. ACE2 was expressed at a similar height to the luminal portion of S protein-positive cells from the basal lamina (Figure 3A). The superficial portion of CK18-positive cells was located lower than the ACE2 signal level (Figure 3B). The superficial portion of OMP-positive cells was located above the luminal surface of S protein-positive cells (Figure 3D). These results are summarized in Figure 3G. We also colocalized SARS-CoV-2 with olfactory cellular components. We were able to colocalize SARS-CoV-2 proteins with CK18 but not with OMP and DCX (Figure 3C–E). Additionally, we colocalized the viral S protein with ChAT-positive cells (Figure 3F).

In the VSE, we also colocalized viral proteins with markers of epithelial components, using the same method as that for the MOE (Figure 4). We found that SARS-CoV-2 proteins were colocalized with CK18 and ChAT, but not with OMP and DCX, which was similar to the results of MOE (Figure 4A–D). Moreover, S proteins encircled not only the nuclei of Sox2-positive cells but also the Sox2-negative cells (Figure 4E).

### 3.3. Pathologic Changes in the Olfactory Epithelium and Its Regeneration

**Figure 5:**
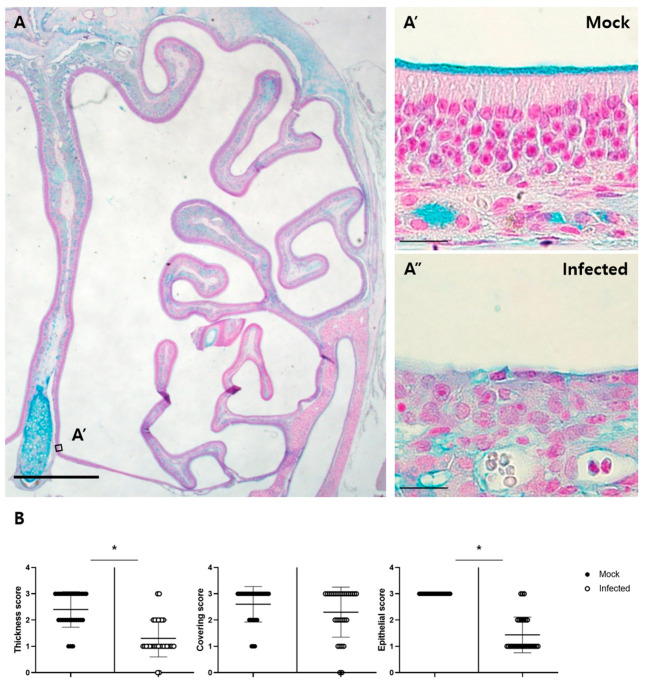
Pathologic changes in the MOE. (**A**) AB-PAS staining in the MOE of the mock group. A’ and A” are representative images cropped from the same region of the mock and infected groups, respectively. Alcian blue-positive mucus materials are seen on the luminal surface of the MOE and Bowman’s glands. The scale bar in (**A**) is 1 mm, and the scale bars in the cropped images are 20 µm; (**B**) histopathological scoring of thickness, mucus covering, and epithelial damage. The scores for mucus thickness and epithelial damage were significantly different between the mock and infected groups (* *p* < 0.05, Mann–Whitney U test, *n* = 4).

Alcian blue staining was performed for the histopathological assessment of olfactory structures (Figure 5A). In the mock-treated group, the Bowman’s glands secreted Alcian blue-positive materials and covered the luminal surface of MOE. The mean thickness score was 2.4. However, in the infected group, the mean thickness score was 1.3, showing a significant reduction (*p* < 0.0001). The mean covering score was 2.6 in the mock group and 2.3 in the infected group, but these scores were not significantly changed. In addition to the mucus changes, the infected epithelium had an abnormal structure. Most of these lost three distinct layers. Some detached epithelium was found in the lumen as cell debris. The mean epithelial score was 3 in the mock group and 1.43 in the infected group, which was significantly reduced (Figure 5A,B). In the VSE, there were no severe lesions, as was also observed in the MOE.

**Figure 6:**
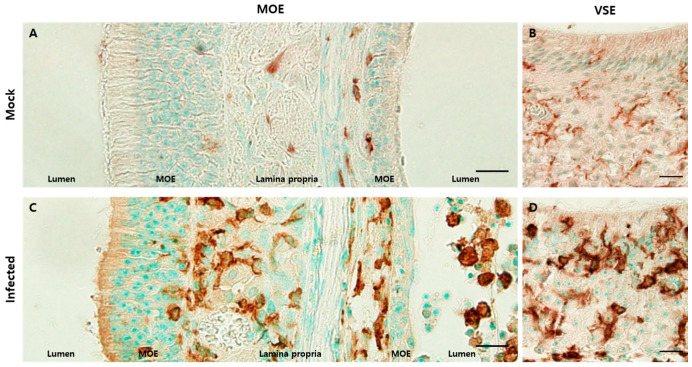
Inflammatory cells infiltrated olfactory structures. Scale bars = 20 µm. (**A**) DAB staining with the antibody against Iba1 in the MOE of the mock group. Iba1-positive cells were mostly present in the lamina propria and some were adjacent to the basal lamina; (**B**) DAB staining with the antibody to Iba1 in the VSE of the mock group. Iba1-positive cells showed their processes, which meant that they were in a resting state; (**C**) DAB staining with the antibody against Iba1 in the MOE of the infected group. There were increased Iba1-positive cell levels. These cells were located in the lamina propria, a relatively superficial portion of the epithelia, and the lumen; (**D**) DAB staining with the antibody to Iba1 in the VSE of the infected group. Many Iba1-positive cells were amoeboid in shape and more densely stained. They were in an active state.

To evaluate immune cell infiltration in olfactory structures, we used an antibody against ionized calcium adaptor molecule 1 (Iba1), a microglia/macrophage-specific calcium-binding protein, as previously described [13]. Iba1-positive monocytes/macrophages were adjacent to the lamina propria in the mock group (Figure 6A). However, in the infected group, they were also found in the lumen and in the upper portion of the epithelium with increased immunoreactivity (Figure 6C). In the VSE, Iba1-positive monocytes/macrophages were in a resting state, expressing their processes in the mock group. By contrast, iba1-positive monocytes/macrophages were amoeboid in shape and densely distributed in the infected group (Figure 6B,D).

**Figure 7:**
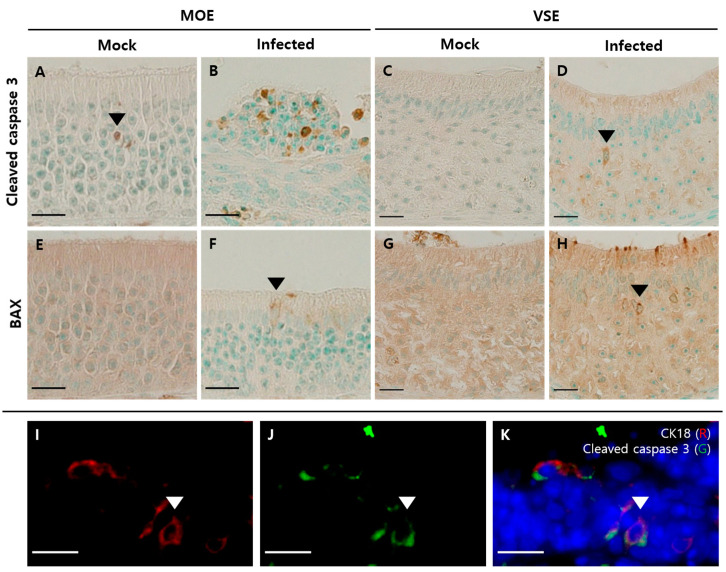
Apoptosis in the olfactory system. Scale bars = 20 µm. (**A**) DAB staining with the antibody against cleaved caspase 3 in the MOE of the mock group. Few cleaved caspase 3-positive cells were observed in the intermediate region of the MOE; (**B**) DAB staining with the antibody against cleaved caspase 3 in the MOE of the infected group. Increased cleaved caspase 3-positive cells were present in various layers of the MOE and cell debris; (**C**) DAB staining with the antibody against cleaved caspase 3 in the VSE of the mock group. There were few cleaved caspase 3-positive cells; (**D**) DAB staining with the antibody against cleaved caspase 3 in the VSE of the infected group. The nuclei of cleaved caspase 3-positive cells appeared as the nuclei of SCs; (**E**) DAB staining with BAX antibody in the MOE of the mock group. There were few BAX-positive cells in the MOE; (**F**) DAB staining with the antibody against BAX in the MOE of the infected group. The nucleus of BAX-positive cells was in the apical layer and resembled the nuclei of SCs; (**G**) DAB staining with the antibody against BAX in the VSE of the mock group. There were few BAX-positive cells in the VSE; (**H**) DAB staining with the BAX antibody in the VSE of the infected group. The nucleus of BAX-positive cells was in the apical layer and resembled the nuclei of SCs. (**I**–**K**) Double immunofluorescence with the antibody against CK18 (red) and cleaved caspase 3 (green) in the MOE of the infected group. Sections were counterstained with DAPI (blue). Some CK18-positive cells colocalized with cleaved caspase 3.

Next, to evaluate the apoptosis in olfactory structures, we used antibodies against cleaved caspase 3 and Bcl-2-associated X (BAX). There were few cleaved caspase 3-positive cells, mostly in the middle layer of the MOE in the mock group (Figure 7A). In the MOE of the infected group, however, we identified increased cleaved caspase 3-positive cells with various distributions, including in the apical layer of the epithelium and lumen (Figure 7B). BAX-positive cells were not found in the MOE of the mock group (Figure 7E). However, in the MOE of the infected group, BAX-positive cells were observed, of which the nucleus was in the apical layer (Figure 7F). In the VSE, we found increased cleaved caspase 3- and BAX-positive cells in the infected group (Figure 7D,H) compared to the mock group (Figure 7C,G). Their nuclei were in the apical layer, and their shapes resembled those of SCs. Next, we performed dual immunofluorescence analysis using cleaved caspase 3 and CK18. We were able to colocalize CK18-positive cells with cleaved caspase 3 in the MOE of the infected group (Figure 7I–K).

**Figure 8:**
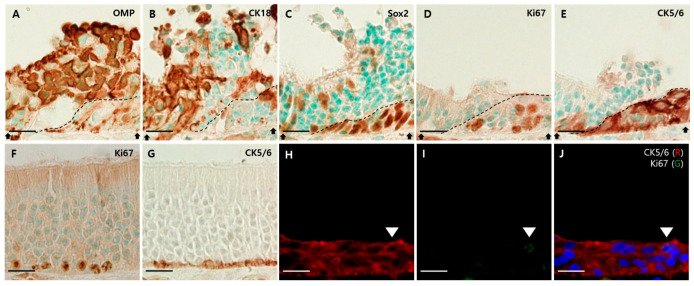
Regeneration of the MOE after viral infection. Scale bars = 20 µm. (**A**) DAB staining with the antibody against OMP in the MOE of the infected group. OMP-positive cells are abundant in the damaged epithelium, but rare in the regenerating epithelium; (**B**) DAB staining with the antibody against CK18 in the MOE of the infected group. CK18-positive cells were abundant in the damaged epithelium, but rare in the regenerating epithelium; (**C**) DAB staining with the antibody against Sox2 in the MOE of the infected group. Sox2-positive cells were in the apical and basal layers of the damaged epithelium. In the regenerating epithelium, Sox2-positive cells formed multilayers; (**D**) DAB staining with the antibody against Ki67 in the MOE of the infected group. Ki67-positive cells formed multilayers in the regenerating epithelium; (**E**) DAB staining with the antibody against CK5/6 in the MOE of the infected group. CK5/6-positive cells formed multilayers in the regenerating epithelium; (**F**) DAB staining with the antibody against Ki67 in the MOE of the mock group. Ki67-positive cells were present in the basal layer of the MOE; (**G**) DAB staining with the antibody against CK5/6 in the MOE of the mock group. CK5/6-positive cells were present in the basal layer of the MOE. They formed a single layer. (**H**–**J**) Double immunofluorescence with antibodies against CK5/6 (red) and Ki67 (green). Sections were counterstained with DAPI (blue). In the superficial layer of the multilayer formed by CK5/6, both CK5/6- and Ki67-positive cells were observed.

Finally, we found evidence of MOE regeneration in the infected group. Here, we focused on the junction between the damaged and regenerating epithelium. Their borders are indicated by a dotted line (Figure 8A–E). The OMP-positive cells and CK18-positive cells were abundant in damaged epithelia, but there were few in regenerating epithelia (Figure 8A,B). The many cells that were composed of regenerating epithelium included Sox2-, Ki67-, and CK5/6-positive cells (Figure 8C–E). Compared to the mock group, Ki67- and CK5/6-positive cells were located only in the basal layer (Figure 8F,G), and Ki67- and CK5/6-positive cells in the infected group spanned the entire regenerating epithelium and formed a multilayer (Figure 8D,E). Additionally, we observed both CK5/6- and Ki67-positive cells in the superficial layer using dual immunofluorescence (Figure 8H–J).

## 4. Discussion

Our results demonstrated that SARS-CoV-2 infects the cellular components of olfactory structures, including MOE and VNO, and indirectly suggested the potential mechanisms of olfactory dysfunction and regeneration in patients with COVID-19, by observing histopathological changes in the olfactory structures of Syrian hamsters.

Previous studies regarding SARS-CoV-2 infection in the olfactory system have used nasal samples from mice, hamsters, and humans (Table 3). Similar results were observed for ACE2 expression and SARS-CoV-2-infected cells in Syrian hamsters, but there were several differences as well. First, we found different ACE2 expression patterns in the MOE. ACE2 expression was found in the apical surface of the epithelium and luminal surface of Bowman’s glands and ducts, as in other studies [11,12,28]. Expression on the apical surface of the epithelium was particularly strong in the dorsomedial region, moderate in the ventromedial region, and weak in the lateral region. Unlike other studies in which ACE2 expression disappeared abruptly [11,12], we observed weak and sparse expression of ACE2 in the ventral region. This difference may be because of differences in the sectional level of the nasal cavity or experimental animals, but further studies are required. Additionally, we concluded that in the MOE, CK18-positive SCs express ACE2 but not OSNs, in a colocalization study.

Second, we identified other SARS-CoV-2 targets. Most previous studies have focused on cells that occupy most of the epithelium, OSNs, and SCs. Here, we observed that SARS-CoV-2 infected ChAT-positive cells, the minor component of olfactory structures, together with CK18-positive cells in the nasal cavity. Although we did not colocalize ChAT-positive cells with ACE2 receptor, other studies revealed that MCs, which are ChAT-positive, have transcripts of the ACE2 and TMPRSS2 genes at a low level [11,30]. These findings support the hypothesis that SARS-CoV-2 infects ChAT-positive cells. In addition, we expect that these findings will help expand the current understanding of olfactory loss. To date, the reasons for olfactory disorders have been classified into the following five mechanisms: obstructive inflammation in olfactory clefts, indirect inhibition of OSN function by inflammatory mediators, direct infection of OSNs, altered microenvironment of MOE caused by the dysfunction of cellular components, and viral infiltration to the brain, resulting in the disturbance of olfactory centers [6,31]. Our results regarding the infection of CK18- and ChAT-positive cells can explain the dysfunction of the MOE microenvironment. CK18-positive SCs are non-neuronal glial-like cells that contribute to the integrity of the MOE, enhancing OSN function, detoxifying xenobiotics, degrading odorants, regulating ionic balance, and engulfing degenerated OSNs [18,24]. Here, we observed not only the infection but also the apoptosis of SCs by colocalizing CK18-positive cells with cleaved caspase 3. The infection and apoptosis of SCs may affect the function of OSNs, as often described in prior studies [31]. In addition to SCs, MCs contribute to olfactory epithelium function. MCs are cholinergic cells that express ChAT and the vesicular acetylcholine transporter. The acetylcholine secreted from MCs affect SCs and OSNs via muscarinic receptors [25,30,32]. MCs are also considered to play a protective role in maintaining the olfactory function of the MOE [33]. Thus, infection of MCs can also be one of the factors causing malfunctions in OSNs.

Solitary chemosensory cells (SCCs), other ChAT-positive cells in the VNO, and the non-olfactory epithelium of the nasal cavity were also found to be infected with SARS-CoV-2 in this study. The S protein encircled not only Sox2-positive cells but also Sox2-negative cells, which means that there are targets of viruses other than SCs. In addition, we were able to colocalize S protein with ChAT-positive cells. SCCs expressed chemosensory signaling molecules similar to MCs, including transient receptor potential channel M5 and G-protein-associated α-gustducin [25]. These are also cholinergic cells, such as MCs. One of the differences between SCCs and MCs is that most SCCs are innervated by peptidergic nociceptive nerve fibers of the trigeminal nerve. When SCCs sense chemical stimuli, they release acetylcholine, resulting in activation of the trigeminal nerve, reflexes, and neurogenic inflammation in the nasal cavity [25,32,34,35,36]. Therefore, infection of these cells may affect the functions of the nasal cavity other than olfaction. Moreover, the possibility that trigeminal nerve fibers can be used as a route to the brain by SARS-CoV-2 cannot be overlooked. Although intraepithelial trigeminal nerve fibers are rare in the MOE [32], there are also sparse innervations of MCs by the trigeminal nerve [25]. It is uncertain whether the terminal fibers of the trigeminal nerve express ACE2 and TMPRSS2. However, considering the presence of viral RNA copies of SARS-CoV-2 in some human trigeminal ganglion samples [29] and neurovirulent murine coronaviruses that infect and spread in the brain via the olfactory and trigeminal nerves [37,38], more research is needed to verify whether SARS-CoV-2 uses the trigeminal nerve as a route to the brain.

From our results, it is possible that there are other candidates that are responsible for olfactory loss, other than SCs and MCs. First, we focused on Bowman’s glands. The mucus produced by Bowman’s glands is necessary for odor detection because it allows odorants to diffuse to olfactory receptors [31,39]. Using Alcian blue stain, which is utilized for mucin staining, we found that there was reduced thickness of Alcian blue-positive mucus on the surface of the infected MOE. Based on these findings, we suspect that insufficient covering of epithelia contributes to olfactory dysfunction, but further studies are needed to clarify whether these changes are due to the dysfunction of Bowman’s glands or epithelial destruction. Second, structural instability may contribute to olfactory dysfunction. As in previous studies, most of the epithelium was desquamated, some cellular debris was present in the lumen [10,21,40], and three distinct layers were lost. Because SCs are responsible for epithelial stability, their infection and subsequent apoptosis may result in these changes. Third, we observed inflammation in the olfactory epithelia. Immunostaining with antibodies against Iba1 indicated an increase in the infiltration of Iba1-positive monocytes/macrophages in the nasal cavity and concha. They were variously distributed in the epithelium and lamina propria. These results corresponded with those of a previous study [21]. Because inflammatory cytokines that are secreted from immune cells can lower the expression of odorant receptor genes indirectly [10,31], studies have suggested inflammation as a possible cause, and our results also support this.

To the best of our knowledge, no study involving the exact regenerative procedure after viral infection has been made so far. Owing to its capacity for neurogenesis, MOE can reconstitute its structure after injury [26]. There are two types of BCs, and their proliferation and differentiation depend on the degree of injury. Globose BCs (GBCs) proliferate during normal ongoing neurogenesis or reconstitution after mild injury. By contrast, horizontal BCs (HBCs) are dormant during normal ongoing neurogenesis and respond to only severe epithelial injuries [41,42]. In the present study, regenerating epithelia were mostly composed of Sox2-, Ki67-, and CK5/6-positive cells, which form multilayers and span the full height of the epithelium, unlike the mock group. Sox2 is expressed in the nucleus of SCs and some BCs, including HBCs and early stages of GBCs. When the MOE is injured, the population of Sox2-negative GBCs, just before becoming iOSNs, return to upstream Sox2-positive GBCs. These are multipotent, and can become OSNs as well as SCs [42]. Therefore, Sox2-positive cells in the regenerating epithelium may regenerate either GBCs, HBCs, or newly generated SCs. Additionally, multiple layers of CK5/6-positive cells and co-expression of CK5/6 with Ki67 indicate that MOE is so severely damaged that it is sufficient to activate the proliferation of HBCs [41,42,43]. In addition, the death of SCs can contribute to the proliferation and activation of the multipotency of HBCs [42]. As we only observed the regenerating epithelium at 4 dpi, which is just the initial point and does not reflect the overall progress, more studies over this time point are needed to understand the whole regenerative procedure of injured epithelium. 

We also investigated ACE2 expression, viral infection, and pathologic changes, including inflammation and apoptosis in the VNO, another olfactory structure responsible for pheromone detection. An interesting fact is that the expression pattern of S protein was different between MOE and VSE. This difference may be due to a difference in the distribution of organelles. During viral replication, translated structural proteins including spike are inserted into the endoplasmic reticulum–Golgi intermediate compartment (ERGIC) before the formation of mature virion [44]. Therefore, if there is a difference in the distribution of ERGIC between infected cells in MOE and VSE, the expression pattern of S protein can be different. The prior study investigating the ultrastructure of MOE and VSE using mice revealed that SCs of VSE have much less cytoplasmic complexity, smaller amounts of endoplasmic reticulum, fewer and smaller Golgi complexes, and fewer dense vacuoles at bases than SCs of MOE, which were suspected as major targets of virus in this study [45]. These ultrastructural differences may influence the expression pattern of S protein in MOE and VSE. Unlike S protein, the expression pattern of N protein would be less affected by the distribution of the organelles because the major role of N protein is packing viral genome into helical ribonucleoprotein complex in cytoplasm, not in organelles [44,46]. This hypothesis is consistent with our result, observing a similar expression pattern of N protein between MOE and VSE.

In VSE, there were no lesions as severe as the MOE. However, activated Iba1-positive monocytes/macrophages, similar to a previous study that found activation of monocytes/macrophages in the MOE [21], and cleaved caspase 3- or BAX-positive apoptotic cells were evident. Considering the function of the VNO, infection and subsequent pathologic changes may affect the behavior of Syrian hamsters. However, it is uncertain whether these results can also be applied to humans, due to rudimentary changes in the VNO of adult humans [47,48,49].

In the present study, we focused on the main receptor of SARS-CoV-2, ACE2. Recently, the neuropilin-1 (NRP-1) has been discovered as an alternative receptor of SARS-CoV-2 [50,51,52]. Furthermore, NRP-1 was highly expressed in infected olfactory epithelial cells in a prior study [51]. Therefore, there is the possibility that SARS-CoV-2 uses NRP-1 to infect OSNs and transfer to the brain. We could not find infection of OSNs, but we observed the olfactory system only at 4 dpi. At the later time point, SARS-CoV-2 may bind to NRP-1, infect OSNs, and transfer to the brain, and this scenario can explain the neuroinvasiveness of SARS-CoV-2 [52]. In addition, further studies are needed to investigate whether other minor cellular components, such as SCCs and MCs, express NRP-1 or not.

In summary, the olfactory structures of Syrian hamsters, including MOE and VNO, expressed ACE2, which is needed for SARS-CoV-2 to enter cells. In the MOE, the virus infected ACE2-expressing SCs. Viral infection results in mucus changes, epithelial detachment, infiltration of inflammatory cells, and apoptosis of cellular components. These structural and functional pathologic changes collectively may explain the causes of olfactory dysfunction, including anosmia, in patients with COVID-19. Injured epithelia are regenerated because of their ability to induce neurogenesis. Therefore, if the regenerated epithelium works properly, olfactory dysfunction may not last indefinitely, although some patients suffer from prolonged dysfunction. In the VNO, ACE2 was expressed on NSE, cavernous tissue, and VSE. Similar to the MOE, SCs in the VSE were infected. There were activated monocytes/macrophages in the VSE after infection. Additionally, SCCs and MCs are other targets of the virus, and their infection may contribute to the dysfunction of olfactory structures.

## Figures and Tables

**Table 1:** Primary antibodies used in this study.

Antibody	Species	Dilution	Target	Company	Reference
ACE2	Rabbit	1:200	SARS-CoV-2 receptor	Novusbio	NBP2-67692
Spike	Mouse	1:200	SARS-CoV-2	GeneTex	GTX632604
Nucleocapsid	Rabbit	1:200	SARS-CoV-2	Novusbio	NB100-56576
OMP	Rabbit	1:1000	mOSN and mVSN	Abcam	ab183947
DCX	Rabbit	1:2000	iOSN and iVSN	Abcam	ab18723
SOX2	Rabbit	1:1000	SC and some BC	Abcam	ab97959
Ki67	Rabbit	1:1000	Proliferating cell	Abcam	ab15580
CK5/6	Mouse	1:100	HBC	Dako	M7237
CK18	Mouse	1:1000	SC	Abcam	ab668
ChAT	Rabbit	1:2000	MC and SCC	Abcam	ab178850
Iba1	Rabbit	1:1000	Monocyte and macrophage	Wako	019-19741
BAX	Mouse	1:200	Apoptotic cell	Santa Cruz	sc-7480
Cleaved caspase 3	Rabbit	1:1000	Apoptotic cell	Abcam	ab2302

ACE2, angiotensin-converting enzyme 2; BAX, Bcl-2-associated X protein; BC, basal cell; ChAT, choline acetyltransferase; CK5/6, cytokeratin 5/6; CK18, cytokeratin 18; DCX, doublecortin; HBC, horizontal basal cell; Iba1, ionized calcium binding adaptor molecule 1; iOSN, immature olfactory sensory neuron; iVSN, immature vomeronasal sensory neuron; mOSN, mature olfactory sensory neuron; mVSN, mature vomeronasal sensory neuron; OMP, olfactory marker protein; SARS-CoV-2, severe acute respiratory syndrome coronavirus 2; SC, supporting cell; MC, microvillar cell; SCC, solitary chemosensory cell; SOX2, SRY-box 2.

**Table 2:** Secondary antibodies used in this study.

Antibody	Species	Company	Reference
Biotinylated anti-mouse IgG	Horse	Vector	BA-2000
Biotinylated anti-rabbit IgG	Horse	Vector	BA-1100
Texas red conjugated anti-mouse IgG	Horse	Vector	TI-2000
FITC-conjugated anti-rabbit IgG	Goat	Santa Cruz	sc-2012

**Table 3:** Previous studies regarding SARS-CoV-2 infection in the main olfactory epithelium.

Reference	ACE2	TMPRSS2	SARS-CoV-2
Anna Jinxia Zhang et al. (2020) [10]	In the apical and middle layer (immunostaining in Syrian hamsters)	-	SCs, iOSNs, and mOSNs (immunostaining in Syrian hamsters)
Leon Fodoulian et al. (2020) [11]	SCs (single cell RNA sequencing in humans, immunohistochemistry in mice and humans)	SCs (single cell RNA sequencing in humans, immunohistochemistry in mice and humans)	-
David H. Brann et al. (2020) [12]	SCs and BCs (bulk and single cell RNA sequencing and immunostaining in mice and humans)	SCs and BCs (bulk and single cell RNA sequencing in mice and humans)	-
Katarzyna Bilinska et al. (2020) [13]	SCs (immunostaining in mice)	SCs and iOSNs (ISH)	-
Rafal Butowt and Katarzyna Bilinska (2020) [14]	Non-neuronal cells (RNA-seq in mice and humans)	Neuronal and non-neuronal cells (RNA-seq in mice and humans)	-
Chen et al. (2020) [15]	SCs (immunostaining in humans)	-	-
Ueha et al. (2020) [16]	SCs, OSNs, and BCs (immunostaining in mice), OSNs (immunostaining in humans)	SCs (immunostaining in mice)	
Betrand Bryche et al. (2020) [21]	-	-	SCs (immunostaining in Syrian hamsters)
Sin Fun Sia et al. (2020) [22]	-	-	OSNs (immunostaining in Syrian hamsters)
Klingenstein et al. (2020) [28]	SCs (immunostaining in humans)	SCs (immunostaining in humans)	-
Jenny Meinhardt et al. (2020) [29]	-	-	Neural/neuronal cells (immunostaining, ISH, and EM in humans)

BC, basal cell; EM, electron microscope; iOSN, immature olfactory sensory neuron; ISH, in situ hybridization; mOSN, mature olfactory sensory neuron; OSN, olfactory sensory neuron; SC, supporting cell.

## Data Availability

The datasets and supporting materials generated during and/or analyzed during the current study are available from the corresponding author on reasonable request.
